# Glutamatergic neurons and GABAergic neurons of medial prefrontal cortex control hoarding-like behavior

**DOI:** 10.3389/fnins.2023.1169927

**Published:** 2023-05-11

**Authors:** Yujie Xiong, Beining Wang, Yunxia Shang, Huan Liu, Zihao Zhan, Qi Xu, Kai Wang, Zhi Zhang, Tingting Sun

**Affiliations:** ^1^Department of Physiology, School of Basic Medical Sciences, Anhui Medical University, Hefei, China; ^2^Department of Neurology, The First Affiliated Hospital of Anhui Medical University, Hefei, China; ^3^Hefei National Laboratory for Physical Sciences at the Microscale, Division of Life Sciences and Medicine, University of Science and Technology of China, Hefei, China

**Keywords:** hoarding disorder, prelimbic cortex, glutamate, GABA, behavior

## Abstract

Hoarding disorder (HD) is a chronic disease that begins early in life and does not remission unless timely treated. A large number of factors affect the presentation of HD symptoms, including a strong possessive psychology of objects and neurocognitive functioning. However, the underlying neural mechanisms of the excessive hoarding behavior in HD are still unknown. Using viral infections and brain slice electrophysiology recordings, we found that increased glutamatergic neuronal activity and decreased GABAergic neuronal activity in medial prefrontal cortex (mPFC) accelerated the hoarding-like behavior in mice. Respectively, chemogenetic manipulation to reduce glutamatergic neuronal activity or enhance GABAergic neuronal activity could improve the hoarding-like behavioral response. These results reveal a critical role played by alterations in the activity of specific types of neurons in hoarding-like behavior, and that targeted therapies for HD may be possible by precisely modulating these types of neurons.

## 1. Introduction

Hoarding disorder (HD) is a relatively common psychiatric disorder characterized by excessive acquisition of, and inability to discard items, regardless of their real value, resulting in clutter as well as significant distress (Frost and Hartl, 1996). Pathological and empirical studies of hoarding behavior are generally poorly designed, and prove extremely challenging for the exploration of behavioral mechanisms.

HD is frequently viewed as an adult disorder, but symptoms of hoarding behavior often appear in childhood and adolescence, which is a new addition to the *Diagnostic and Statistical Manual of Mental disorders*, Fifth Edition (DSM-5; Mataix-Cols et al., 2010; Regier et al., 2013). Although previously considered one of the symptoms of obsessive–compulsive disorder (OCD; Winsberg et al., 1999), compared to other subtypes of OCD, hoarding behavior has been associated with relatively poor response in cognitive–behavioral therapy (CBT; Mataix-Cols et al., 2002) and exhibits cognitive deficits in the value judgment of possessions, including difficulties in deciding to discard objects and classifying them (Frost and Hartl, 1996; Woody et al., 2014). Furthermore, hoarding symptoms may be actually more common than OCD (Samuels et al., 2008; Stein et al., 2019), with over 80% of hoarding adults not meeting the diagnosis criteria of OCD (Frost et al., 2011) and showing fewer symptoms of anxiety (Burton et al., 2016). Thus, HD is distinct from OCD in the DSM-5 and its etiology and pathology are still uncertain.

The medial prefrontal cortex (mPFC) is associated with decision making (Euston et al., 2012), including detection errors (Holroyd et al., 2002) and cognitive control (Ridderinkhof et al., 2004), which is divided into anterior cingulate cortex (ACC), prelimbic cortex (PrL), and infralimbic cortex (IL), based on functional and anatomical characteristics (Heidbreder and Groenewegen, 2003; Sparta et al., 2014). As a cortical region, mPFC is made up of different neuronal types and projections, most of which are excitatory glutamatergic neurons, and a smaller proportion are inhibitory GABAergic neurons (Xu et al., 2019). GABAergic neurons can be categorized into multiple distinct classes, such as somatostatin (SST)-expressing GABAergic neurons and parvalbumin (PV)-expressing GABAergic neurons. SST neurons locally innervate nearby most glutamatergic neurons, and genetic methods regulated the activity of these neurons in mice, revealing a crucial role in the learning and decision making state (Urban-Ciecko and Barth, 2016). *In vivo* animal models demonstrate that the mPFC lesioned mice hoard fewer pellets from an external tube to the home base (Deacon et al., 2003). Hoarding is a systematic process of movement that involves going to the items source, picking, transporting, and dropping it down somewhere else (Deacon, 2006). These schemas typically refer to many brain regions, such as the mPFC and the hippocampus. Therefore, it is not surprising that lesions of both structures weaken hoarding behavior (Kolb, 1974; Deacon et al., 2002). However, causal relationship between these areas and the underlying mechanism of hoarding is unclear.

Combining immunostaining, brain slice electrophysiology and viral injection methods, we dissected the functional organization of the mPFC neurons in the hoarding mice, focusing on inhibitory neurons. Chemogenetic manipulation of the glutamatergic neurons and GABAergic neurons of mPFC, respectively, demonstrated that alterations in the activity of these two types of neurons are necessary to modulate hoarding-like behavior. Notably, we identified subtypes of inhibitory neurons and found that SST neurons, not PV neurons, play an essential role in the hoarding state.

## 2. Materials and methods

### 2.1. Animals

Female C57BL/6 J (aged 4 weeks) mice were purchased from Beijing Vital River Laboratory Animal Technology Co., Ltd., China. All animal experimental procedures were approved by the Institutional Animal Care and Use Committee of Anhui Medical University. Mice were housed in groups of four to five animals per cage under a 12-h light/dark cycle (8:00 a.m./8:00 p.m.) with food and water *ad libitum*, and at a stable room temperature (23°C–25°C). Groups were randomized for all experiments.

### 2.2. Drugs

Clozapine *N*-oxide (TOCRIS) was dissolved in physiological saline (0.33 mg/mL) and injected intraperitoneally. Equivalent volumes of saline were used for control injections. 1 μM tetrodotoxin (TTX, purchased from Hebei Aquatic Science and Technology Development Company, China), 4 mM Kynurenic acid (KYN; Sigma-Aldrich), 100 μM Picrotoxin (PTX; Sigma-Aldrich) were dissolved in standard ACSF.

### 2.3. Animal model of hoarding-like behavior

**Figure 1:**
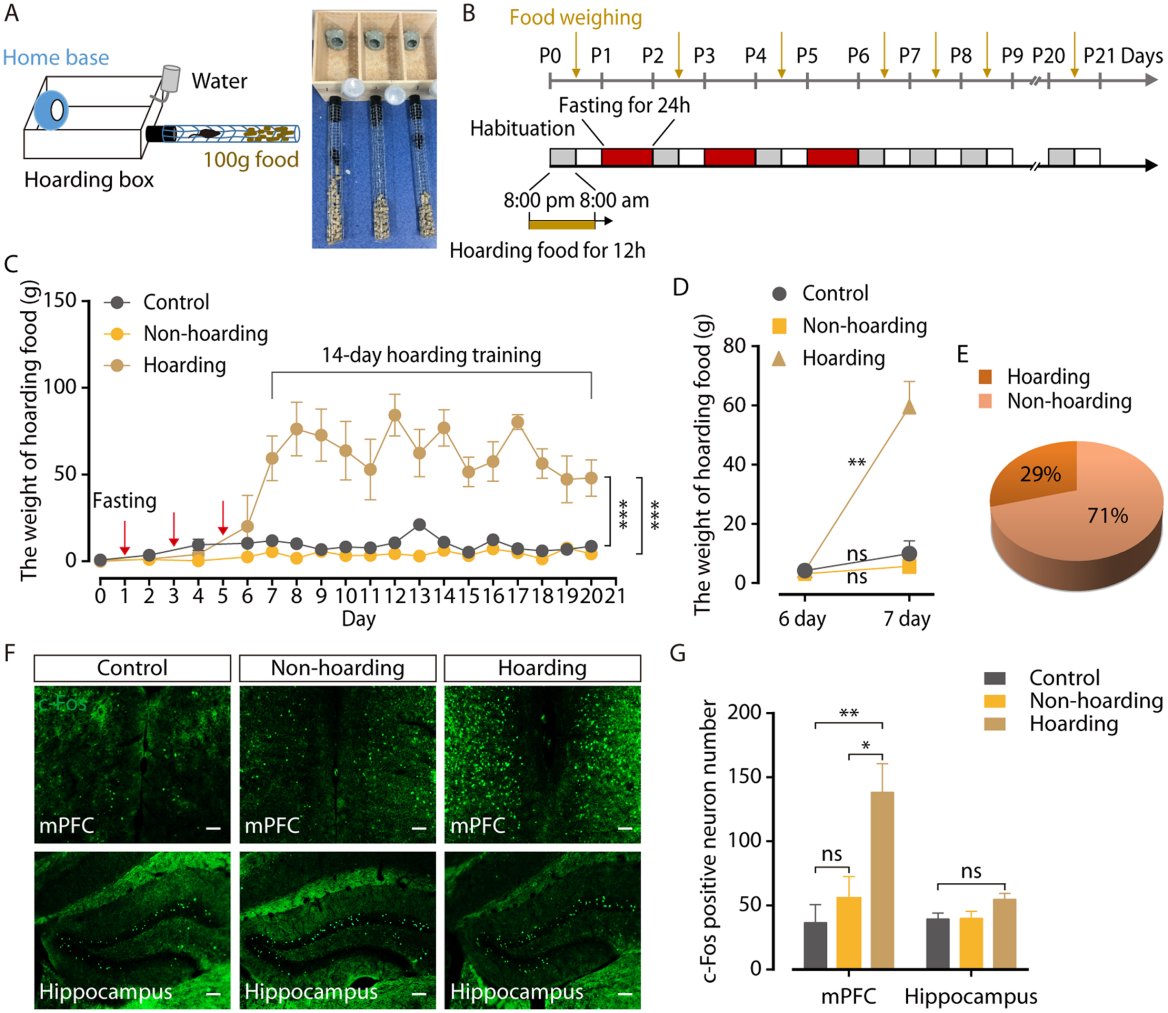
Mouse model of hoarding-like behavior. **(A)** Diagram of hoarding box. **(B)** Timeline for training paradigm. **(C)** The weight of hoarding food of mice treated with fasting 24 h every other day three times (hoarding or non-hoarding) or without (control; *n* = 5–12 mice/group, control vs. hoarding: *F*_1,12_ = 70.65, *p* < 0.0001; non-hoarding vs. hoarding: *F*_1,15_ = 125.3, *p* < 0.0001). **(D)** After termination of fasting, the weight of hoarding food were increased in hoarding mice relative to non-hoarding mice or control mice on day 7 (*n* = 5–12 mice/group, control: t_6_ = −1.45, *p* = 0.198; non-hoarding: t_11_ = −1.84, *p* = 0.092; hoarding: t_4_ = −6.45, *p* = 0.003). **(E)** Distribution of hoarding mice (29%) and non-hoarding mice (71%) in the fasting mice. **(F,G)** Distribution of c-Fos-positive neurons in the mPFC and hippocampus (*n* = 6–10 slices from 3 to 5 mice/group, mPFC, control vs. hoarding: t_14_ = −3.27, *p* = 0.006; non-hoarding vs. hoarding: t_14_ = −2.59, *p* = 0.0215; control vs. non-hoarding: t_10_ = −0.86, *p* = 0.412; hippocampus, control vs. hoarding: t_13_ = −2.07, *p* = 0.059). Average c-Fos-positive neurons per 1.2 mm^2^ imaging area. (Scale bars: 100 μm.) Data are means ± SEM. ^*^p < 0.05, ^**^p < 0.01, ^***^p < 0.001. ns, not significant. Two-way repeated-measures ANOVA with Bonferroni *post hoc* analysis for **(C)**; paired *t*-test for **(D)**; unpaired *t*-test for **(G)**.

Hoarding food can maintain normal physiology needs in rodents with obvious survival value. Multiple 24-h fasting activities (three times, every other day) were used to induce hoarding behavior in a mouse model. The behavioral field (Figure 1A) was a wooden hoarding box (30 cm × 13 cm × 15 cm) that contained a 7 cm × 7 cm × 8.5 cm opaque home base (4.5 cm diameter) for mice to rest, with a wire mesh tube (45 cm long, 4 cm diameter). Through a hole in front of the hoarding box at floor level, a black plastic tube (10 cm long, 4 cm diameter) was inserted to connect the wire mesh tube (Deacon, 2006). Hoarding behavior was measured by weighing food that transported from the wire mesh tube into the hoarding box.

In the training paradigm, mice were first put into the behavioral field from 8:00 p.m. (light off) to 8:00 a.m. (light on) next morning to acclimate to its new surroundings and hoard food. At 8:00 a.m. of the first day, the mice were taken out of the behavioral field and placed back in their cages. The food that transported from the wire mesh tube into the hoarding box was weighed. From 8:00 p.m. on the first day to 8:00 p.m. on the second day, mice were fasted for 24 h in their cages. Mice were placed again into the behavioral field at 8:00 p.m. of the second day until they were taken out at 8:00 a.m. on the third day, during which food was hoarded. Animals were subsequently fasten for 24 h on days 1, 3, and 5, and then without fasting for 14 days.

### 2.4. Measurement of anxiety-like behavior

#### 2.4.1. Open field test

An open field apparatus was composed of a square area (25 cm × 25 cm) and a marginal area (50 cm × 50 cm × 60 cm). Mice were placed in one of four corners and allowed to explore the surroundings freely. The animals’ locomotion traces were recorded for 5 min by a video camera. Time and the entries in the center area and total distance in the open field were analyzed by EthoVision XT software (Sun et al., 2019).

#### 2.4.2. Elevated plus maze test

The elevated plus maze consisted of a central zone (6 × 6 cm^2^), two opposite closed arms (30 × 6 × 20 cm^3^) and two opposite open arms (30 × 6 cm^2^), elevated 100 cm above the ground. Mice were placed in the central zone facing one of the closed arms and allowed to explore the plus-shaped apparatus for 5 min. The animals’ locomotion traces were recorded by a video camera. The time and entries in the opposite open arms and total distance in the maze were analyzed by EthoVision XT software.

### 2.5. Virus injection

Prior to surgery, mice were anesthetized with xylazine (10 mg/kg) and ketamine (100 mg/kg), and then placed on a stereotaxic apparatus (Zhongshi, Beijing, China). Body temperature was maintained at 36°C with a heating pad. A volume of approximately 200 nL virus was injected to target site at a rate of 40 nL/min, using calibrated glass microelectrode connected to an infusion pump (Zhongshi, Beijing, China). For immunostaining experiments to merge with c-Fos, the rAAV-*GAD67*-mCherry-WPRE-hGH pA (AAV2/9, 5.96 × 10^12^ vg/mL, BrainVTA, China) or rAAV-SST-mCherry-WPRE-bGH polyA (AAV2/9, 5.57 × 10^12^ vg/mL, BrainVTA, China) or rAAV-PV-EGFP-bGH polyA (AAV2/9, 2.93 × 10^12^ vg/mL, BrainVTA, China) virus was injected unilaterally to the medial prefrontal cortex (mPFC) of female C57 mice (A/P, +1.92 mm from bregma; M/L, −0.26 mm; D/V, −1.70 mm from cortex). For chemogenetic manipulation of glutamatergic neurons in the mPFC, the rAAV-*CaMKIIa*-hM3D(Gq)-mCherry-WPREs (AAV2/9, 5.29 × 10^12^ vg/mL, BrainVTA, China) or rAAV-*CaMKIIa*-hM4D(Gi)-mCherry-WPREs (AAV2/9, 5.40 × 10^12^ vg/mL, BrainVTA, China) virus was injected unilaterally to the mPFC of female C57 mice (A/P, +1.92 mm from bregma; M/L, −0.26 mm; D/V, −1.70 mm from cortex). The rAAV-*CaMKIIa*-mCherry-WPRE-hGH pA (AAV2/9, 5.14 × 10^12^ vg/mL) virus was used as the controls. For chemogenetic manipulation of GABAergic neurons in the mPFC, the rAAV-*GAD67*-hM3D(Gq)-mCherry-WPREs (AAV2/9, 2.66 × 10^12^ vg/mL, BrainVTA, China) or rAAV-*GAD67*-hM4D(Gi)-mCherry-WPREs (AAV2/9, 3.40 × 10^12^ vg/mL, BrainVTA, China) virus was injected unilaterally. The rAAV-*GAD67*-mCherry-WPRE-hGH pA (AAV2/9, 5.96 × 10^12^ vg/mL) virus was used as the controls. For chemogenetic manipulation of somatostatin(SST)-expressing GABAergic neurons in the mPFC, the rAAV-fSST-CRE-bGH pA (AAV2/9, 5.36 × 10^12^ vg/mL, BrainVTA, China) and the rAAV-hSyn-DIO-hM3D(Gq)-mCherry-WPRE-hGH pA (AAV2/9, 5.14 × 10^12^ vg/mL, BrainVTA, China) or rAAV-hSyn-DIO-hM4D(Gi)-mCherry-WPRE-hGH pA (AAV2/9, 5.67 × 10^12^ vg/mL, BrainVTA, China) virus were injected unilaterally. The viral expression lasted for 3 weeks after injection. CNO (3 mg/kg, i.p.) was injected and behavioral tests were performed 30 min after injection. After the experimental procedure was completed, all mice were transcardially perfused with 0.9% saline and then with ice-cold phosphate buffer (0.1 M) that contained 4% paraformaldehyde. Images of the virus expression were obtained by a confocal microscope (LSM 880, ZEISS, Germany). Mice with missed injections were excluded.

### 2.6. Brain slice electrophysiology

Animals were deeply anesthetized with xylazine (10 mg/kg) and ketamine (100 mg/kg), and then intracardially perfused with 20 ml ice-cold N-methyl-D-glucamine artificial cerebrospinal fluid (NMDG ACSF) saturated with 95% O_2_ and 5% CO_2_. The NMDG ACSF contained (in mM): 93 N-methyl-D-glucamine (NMDG), 2.5 KCl, 1.2 NaH_2_PO_4_, 30 NaHCO_3_, 20 HEPES, 25 glucose, 2 thiourea, 5 Na-ascorbate, 3 Na-pyruvate, 0.5 CaCl_2_, and 10 MgSO_4_, 3 glutathione (GSH; osmolarity: 300–310 mOsm/kg). The pH of NMDG ACSF was titrated to 7.3–7.4 with concentrated HCl. The mice were quickly decapitated, and brains transferred to the same NMDG ACSF on a VT-1200S vibratome (Leica, Germany). Coronal brain slices (300 μm) were sectioned at 0.14–0.18 mm/s. Slices were initially incubated in NMDG ACSF for 10–15 min at 32°C, followed by N-2-hydroxyethylpiperazine-N-2-ethanesulfonic acid (HEPES) ACSF for at least 1 h at 28°C. The HEPES ACSF contained (in mM): 92 NaCl, 2.5 KCl, 1.2 NaH_2_PO_4_, 30 NaHCO_3_, 20 HEPES, 25 glucose, 2 thiourea, 5 Na-ascorbate, 3 Na-pyruvate, 2 CaCl_2_, and 2 MgSO_4_, 3 glutathione (GSH; pH: 7.4, osmolarity: 300–310 mOsm/kg). Brain slices were transferred into a slice chamber (Warner Instruments, United States) for recording and continuously perfused with standard ACSF at 2.5–3 mL/min at 28°C maintained by an inline solution heater (TC-344B, Warner Instruments, USA). The standard ACSF contained (in mM): 129 NaCl, 3 KCl, 2.4 CaCl_2_, 1.3 MgSO_4_, 1.2 KH_2_PO_4_, 20 NaHCO3, 3 HEPES and 10 glucose (pH: 7.4, osmolarity: 300–310 mOsm/kg). Neurons were visualized on an upright microscopy (BX51WI, Olympus, Japan) using infrared interference contrast video monitor. The signals were acquired by using a MultiClamp 700B amplifier (2.8 kHz low-pass Bessel filter and 10 kHz digitization) with pClamp 10.3 software (Molecular Devices, Sunnyvale, CA, USA). Glass capillaries (VitalSense Scientific Instruments Co., Ltd., Wuhan, China) with resistances 6–8 MΩ were pulled using a micropipette puller (P-1000, Sutter Instruments, United States). The electrophysiological data collected with series resistance (10–30 MΩ) was monitored.

For recording the excitability of neurons, the step currents were injected in the current patch mode. The pipettes filled with intracellular solution containing (in mM): 130 K-gluconate, 2 MgCl2, 5 KCl, 0.6 EGTA, 10 HEPES, 2 Mg-ATP and 0.3 Na-GTP (osmolarity: 290–300 mOsm/kg) and pH adjusted to 7.2 with KOH. Neurons were voltage clamped at −70 mV for recording mEPSCs. Inhibitory postsynaptic currents were blocked with 100 μM PTX and 1 μM TTX in the ACSF. Neurons were voltage clamped at −60 mV for recording mIPSCs, the pipettes filled with intracellular solution containing (in mM): 120 KCl, 30 NaCl, 5 EGTA, 10 HEPES, 1 MgCl2, 0.5 CaCl2 and 2 Mg-ATP (osmolarity: 290–300 mOsm/kg) and pH adjusted to 7.2 with KOH. To abolish excitatory synaptic transmission, 4 mM KYN was applied and 1 μM TTX was added to standard ACSF to block the voltage-gated sodium channel.

### 2.7. C-Fos immunostaining

Animals were placed in the behavioral field for 2 h on day 21 and were deeply anaesthetized with xylazine (10 mg/kg) and ketamine (100 mg/kg) for transcardial perfusion with saline followed by 4% paraformaldehyde (0.1 M PB pH 4.5). Brains were extracted and post-fixed in 4% paraformaldehyde overnight at 4°C and immersed in 30% sucrose in 0.01 M PBS (pH 7.4) for cryoprotection. Coronal sections (40 μm) were obtained with a Frozen Slicer (Leica DM1860). Sections were rinsed in PBS three times for 10 min each time, permeabilized in 0.3% TritonX-100 for 30 min at 37°C, blocked for 1 h with 5% normal donkey serum at room temperature and then incubated with primary antibodies, including anti-c-Fos (1:500, rabbit, Cell Signaling Technology), anti-GABA (1:500, rabbit, Sigma Aldrich), anti-glutamate (1:500, rabbit, Sigma Aldrich) and anti-somatostatin (1:500, rabbit, ab108456), at 4°C for 24 h. Sections were then rinsed in PBS three times for 10 min each time, and incubated with Alexa Fluor 488-conjugated donkey anti-rabbit secondary antibody (1:500, abcam, ab150073) or Alexa Fluor 647-conjugated donkey anti-rabbit secondary antibody (1:500, abcam, ab150075) for 1.5 h at room temperature. Fluorescence staining images were observed under fluorescence microscopy (Leica DM2500).

### 2.8. Statistical analysis

Animals were randomized to experimental groups. Simple statistical comparisons was performed by two-tailed paired or two-tailed unpaired Student’s *t*-test. Two-way repeated-measures ANOVA, and Bonferroni *post hoc* analyses were used to statistically analyze the data from the experimental groups with multiple comparisons. All data are expressed as the mean ± SEM, and significance levels are indicated as ^*^p < 0.05, ^**^p < 0.01, and ^***^p < 0.001. OriginPro 2017 software (OriginLab Corporation, United States) and GraphPad Prism 7 (GraphPad Software, Inc., United States) were used for statistical analyses and graphing. Offline analysis of the data obtained from electrophysiological recordings was conducted using Clampfit software version 10.7 (Axon Instruments, Inc., United States) and MiniAnalysis software version 6.03 (Synaptosoft Inc., United States).

## 3. Results

### 3.1. A mouse model of hoarding-like behavior

To identify the neural mechanisms controlling hoarding-like behavior, we constructed a fasting-induce behavior mouse model (Deacon, 2006). The behavioral field was a wooden hoarding box with an opaque home base for mice to rest, and a wire mesh tube with food pellets inside (Figure 1A). Hoarding behavior was measured by weighing food that transported from the wire mesh tube into the hoarding box. Considering that hoarding symptoms occur in childhood and adolescence, gradually increasing with age, mice were fasten at 1 month of age to induce the food hoarding behavior (Cath et al., 2017; Zaboski et al., 2019). Fasting for 24 h once every other day for three consecutive times (Figure 1B) increased the weight of food hoarded in mice during 14 days of training, compared to mice without fasting (Figure 1C; Supplementary Figure 1A). It was worth noting that after terminating fasting activities, only 29% of the mice in the fasting group significantly hoarded more food on day 7, named as the hoarding mice, which is consistent with the low incidence of hoarding symptoms (Steketee and Frost, 2003; Samuels et al., 2008). Meanwhile 71% of the mice in the fasting group, named as the non-hoarding mice, did not show a significant increase in hoarding food at the same timepoint (Figures 1D,E). Furthermore, the body weight of the mice decreased significantly after fasting (Supplementary Figure 1B), which may be an inducing factor for food hoarding during the 14 days of training. In addition, neither hoarding mice nor non-hoarding mice had significant differences in the open field and the elevated plus maze, compared to control mice (Supplementary Figure 2), indicating that hoarding mice did not show anxiety-like emotional states (Regier et al., 2013; Sun et al., 2019).

To investigate the specific brain regions involved in hoarding-like behavior, we searched for c-Fos protein expression in the mouse brain after 20-day hoarding training (Hoffman et al., 1993). Compared with control mice and non-hoarding mice, the medial prefrontal cortex (mPFC) of the hoarding mice expressed a large amount of c-Fos protein, while the expression of c-Fos in the hippocampus was not significant (Figures 1F,G). These results suggest that the mPFC region plays a role in hoarding-like behavior.

### 3.2. Increased activity of mPFC glutamatergic neurons controls hoarding-like behavior

**Figure 2:**
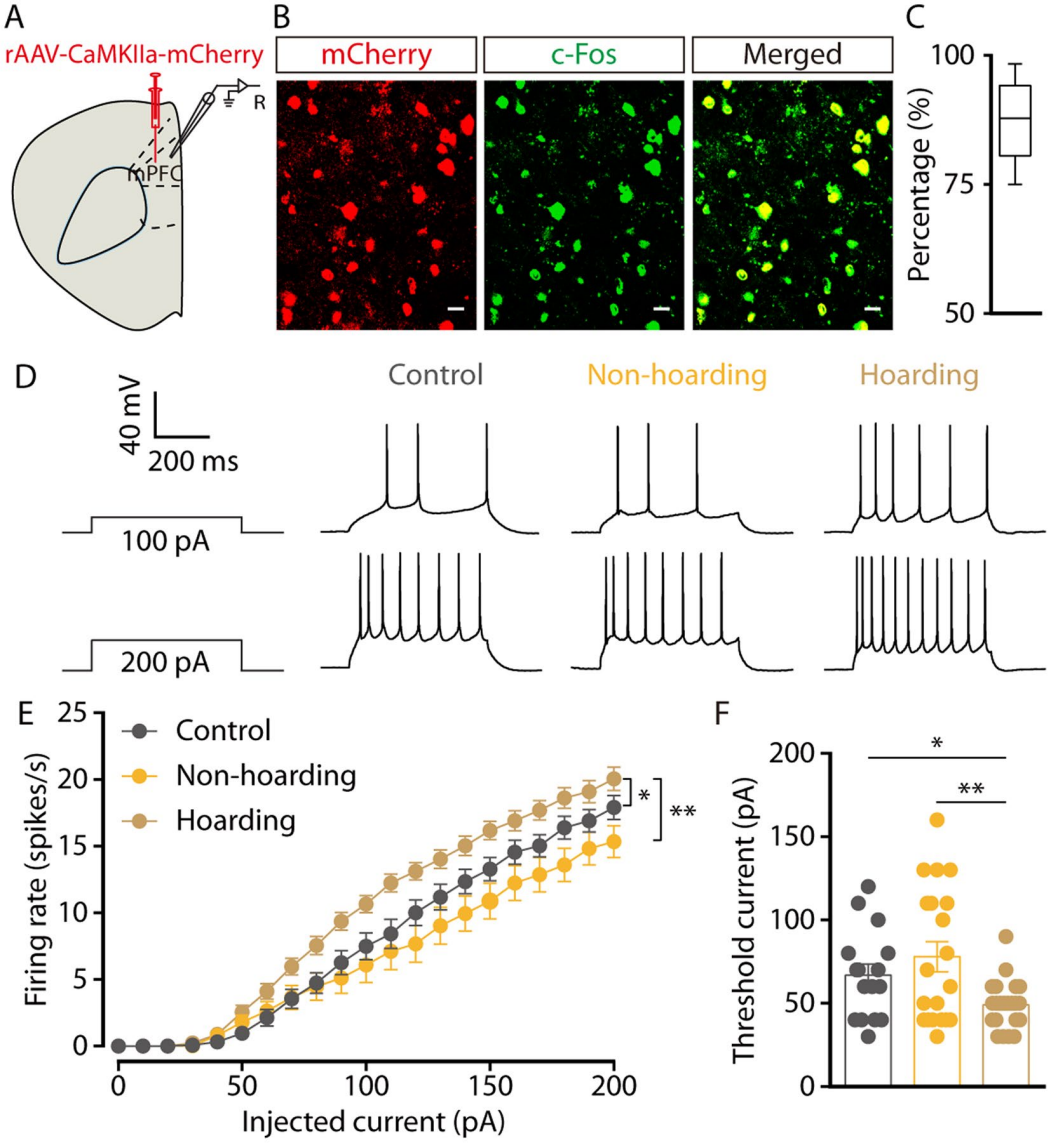
Increased activity of mPFC glutamatergic neurons in hoarding-like behavior mice. **(A)** Schematic diagram for rAAV-*CaMKIIa*-mCherry virus injection and whole-cell recording in slices. **(B)** Example images of mCherry-positive neurons (red) merged with the c-Fos-positive neurons (green) in the mPFC. (Scale bars: 20 μm.) **(C)** Percentage of c-Fos-labeled neurons that expressed *CaMKII* in the mPFC (*n* = 7 slices from 3 mice). **(D)** Representative traces of voltage responses recorded from mCherry^+^ mPFC neurons in slices from mice treated with hoarding training for 20 days. **(E)** Summarized data showing firing rates of evoked action potentials in the groups as indicated in **(D)** (*n* = 16–22 neurons/group, control vs. hoarding: *F*_1,36_ = 6.99, *p* = 0.012; non-hoarding vs. hoarding: *F*_1,41_ = 10.17, p = 0.003; control vs. non-hoarding: *F*_1,35_ = 0.99, *p* = 0.326). **(F)** Statistical data for rheobase recorded from mCherry^+^ mPFC neurons in the groups as indicated in **(D)** (*n* = 16–22 neurons/group, control vs. hoarding: t_36_ = 2.66, *p* = 0.012; non-hoarding vs. hoarding: t_40_ = 3.13, *p* = 0.003; control vs. non-hoarding: t_34_ = −0.95, *p* = 0.351). Data are means ± SEM. ^*^p < 0.05, ^**^p < 0.01. Two-way repeated-measures ANOVA with Bonferroni *post hoc* analysis for **(E)**; unpaired *t*-test for **(F)**.

To determine the activity of mPFC glutamatergic neurons in hoarding mice, whole-cell recordings were used in brain slices from mice with mPFC infusion of the rAAV-*CaMKIIa*-mCherry virus. Three weeks after viral expression, *CaMKII* neurons were visualized during recording (Figure 2A). Co-staining of c-Fos and viral expression in brain slices, showed that mPFC c-Fos^+^ neurons were mostly glutamatergic (87.39% ± 3.25, Figures 2B,C) in the hoarding mice. In response to step current injections, we found an increase in the spike rate (Figures 2D,E) and a decrease in rheobase (Figure 2F) of mPFC mCherry^+^ neurons from hoarding mice compared with those of control or non-hoarding mice. These results indicate an increase in glutamatergic neuronal activity in hoarding states.

**Figure 3:**
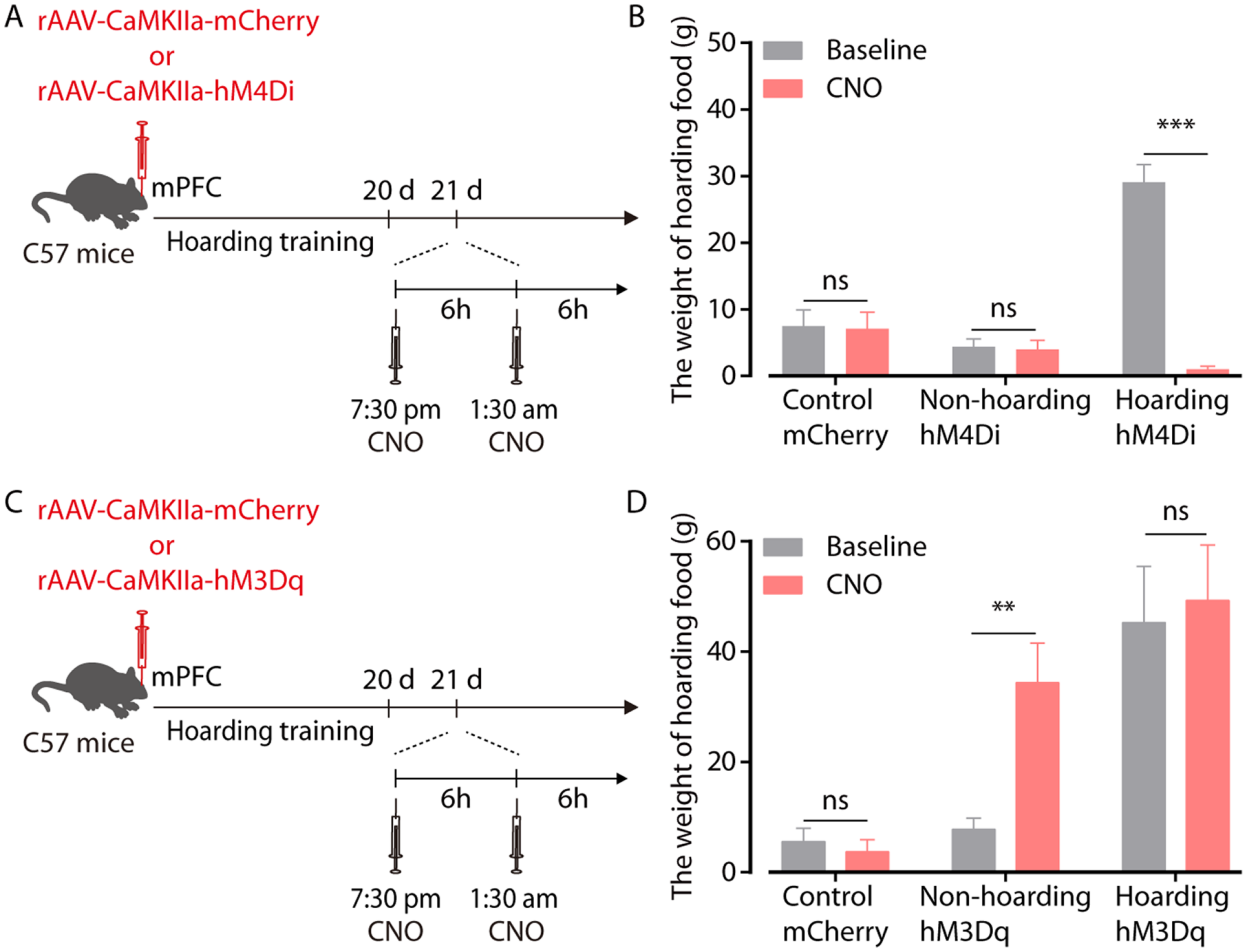
mPFC glutamatergic neuronal activity regulates hoarding-like behavior. **(A)** Schematic of viral injection and chemogenetic manipulation *in vivo*. **(B)** Behavioral effects of the chemogenetic inhibition of mPFC *CaMKII* neurons in non-hoarding and hoarding mice (*n* = 6–12 mice/group, control-mCherry: t_7_ = 0.20, *p* = 0.845; non-hoarding-hM4Di: t_11_ = 0.24, *p* = 0.813; hoarding-hM4Di: t_5_ = 9.98, *p* = 0.0002). **(C)** Schematic of viral injection and chemogenetic manipulation *in vivo*. **(D)** Behavioral effects of the chemogenetic activation of mPFC *CaMKII* neurons in non-hoarding and hoarding mice (*n* = 5–7 mice/group, control-mCherry: t_5_ = 0.51, *p* = 0.632; non-hoarding-hM3Dq: t_6_ = 4.08, *p* = 0.006; hoarding-hM3Dq: t_4_ = 1.52, *p* = 0.202). Data are means ± SEM. ^**^p < 0.01, ^***^p < 0.001. ns, not significant. Paired *t*-test for **(B,D)**.

To investigate the relationship between enhanced mPFC glutamatergic neuronal activity and hoarding-like behavior, we infused rAAV-*CaMKIIa*-hM4Di virus into the mPFC to suppress the activity of glutamatergic neurons in non-hoarding mice and hoarding mice. Considering that the efficacy peaks after intraperitoneal injection of clozapine-N-oxide (CNO) for about half an hour and can be maintained for 6 h, mice were injected twice within 12 h of hoarding food (Figure 3A). We found that chemogenetic inhibition of glutamatergic activity significantly reduced the weight of hoarding food in hoarding mice without reducing the weight of hoarding food in non-hoarding mice and control mice, showing that these manipulations alleviated hoarding-like behavior in mice (Figure 3B; Supplementary Figure 3A). In addition, chemogenetic activation of glutamatergic activity in the mPFC significantly increased the weight of hoarding food in non-hoarding mice, indicating these manipulations accelerated the hoarding-like behavior process in non-hoarding mice (Figures 3C,D; Supplementary Figure 3B). These behavioral consequences confirmed the functional causality of the mPFC glutamatergic activity in the development of hoarding-like behavior.

### 3.3. The mechanism of increased activity of mPFC glutamatergic neurons

**Figure 4:**
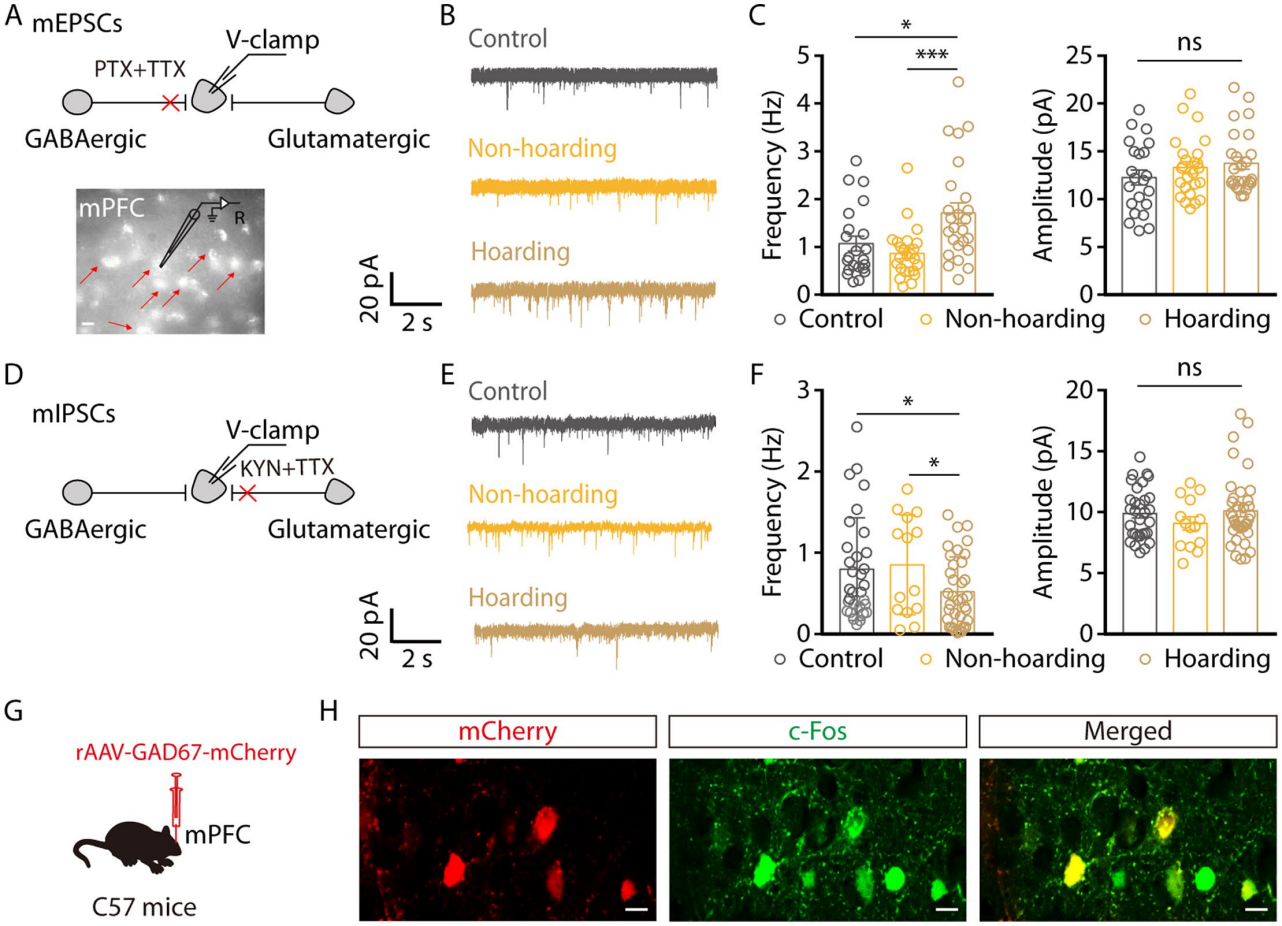
Increased excitatory transmitter input and decreased inhibitory transmitter input of mPFC glutamatergic neurons in hoarding-like behavior mice. **(A)** Schematic of the mEPSCs recorded from mCherry^+^ neurons (indicated by the red arrows) in the mPFC. (Scale bar: 20 μm.) **(B)** Representative traces of mEPSCs in the mPFC slices from mice treated with hoarding training for 20 days. **(C)** Summarized data of the frequency and amplitude of mEPSCs from the groups in **(B)** (*n* = 22–25 neurons/group, frequency, control vs. hoarding: t_45_ = −2.38, *p* = 0.022; non-hoarding vs. hoarding: t_47_ = −3.53, *p* = 0.0009; control vs. non-hoarding: t_44_ = 1.11, *p* = 0.274; amplitude, control vs. hoarding: t_46_ = −1.49, *p* = 0.143). **(D)** Schematic of the mIPSCs recorded from mCherry^+^ neurons in the mPFC. **(E)** Representative traces of mIPSCs in the mPFC slices from mice treated with hoarding training for 20 days. **(F)** Summarized data of the frequency and amplitude of mIPSCs from the groups in **(E)** (*n* = 14–36 neurons/group, frequency, control vs. hoarding: t_65_ = 2.15, *p* = 0.035; non-hoarding vs. hoarding: t_48_ = 2.16, *p* = 0.036; control vs. non-hoarding: t_43_ = −0.26, *p* = 0.799; amplitude, control vs. hoarding: t_65_ = −0.34, *p* = 0.738). **(G)** Schematic diagram for rAAV-*GAD67*-mCherry virus injection. **(H)** Example images of mCherry-positive neurons (red) merged with the c-Fos-positive neurons (green) in the mPFC. (Scale bars: 20 μm.) Data are means ± SEM. ^*^p < 0.05, ^***^p < 0.001. ns, not significant. Unpaired *t*-test for **(C,F)**.

Given that the enhanced activity of mPFC glutamatergic neurons in hoarding-like behavior, we next dissected the mechanism of increased activity through whole-cell recordings. To visualize glutamate neurons in mPFC, mice were injected with rAAV-*CaMKIIa*-mCherry virus. We found that the miniature excitatory postsynaptic currents (mEPSCs) frequency, but not amplitude, of mPFC glutamatergic neurons from hoarding mice was increased (Figures 4A–C). In contrast, the miniature inhibitory postsynaptic currents (mIPSCs) frequency was decreased (Figures 4D–F). These results suggest that enhanced glutamatergic neuronal activity is due to increased excitatory input and decreased inhibitory input. In addition, glutamatergic neurons in the cortex receive strong inhibitory projection, which is supported by a microcircuit organization wherein glutamatergic neurons are innervated by local GABA interneuron (Sun et al., 2019). Therefore, we speculate that cortical inhibitory neurons play an role in hoarding-like behavior, which is supported by c-Fos co-staining with *GAD67* viral expression in brain slices, where some of the mPFC c-Fos^+^ neurons were GABAergic (Figures 4G,H) in the hoarding mice.

### 3.4. Somatostatin-expressing GABAergic neurons In hoarding-like behavior

**Figure 5:**
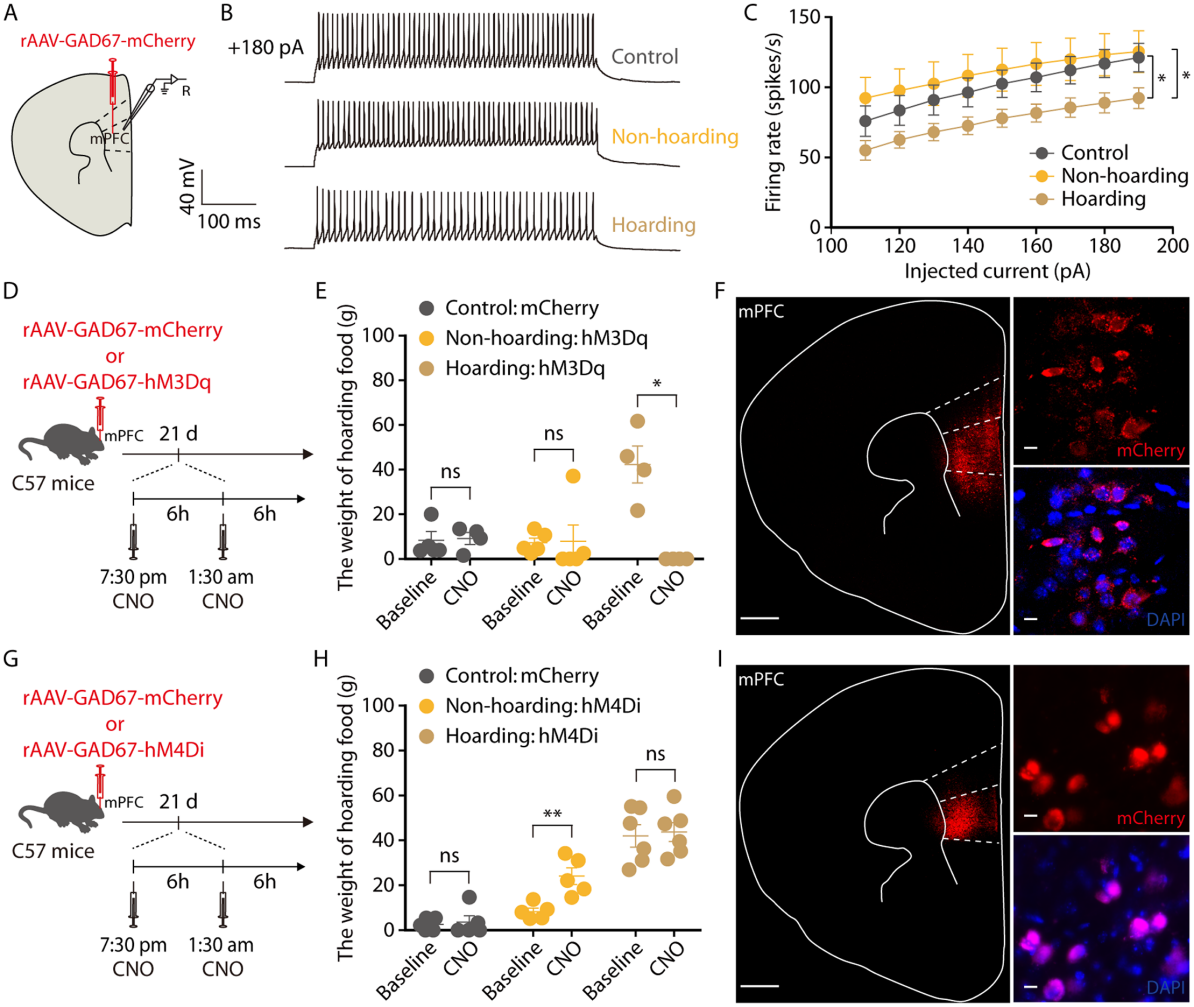
Decreased activity of mPFC GABAergic neurons is involved in hoarding-like behavior. **(A)** Schematic diagram for rAAV-*GAD67*-mCherry virus injection and whole-cell recording in slices. **(B)** Representative traces of voltage responses recorded from mCherry^+^ mPFC neurons in slices from mice treated with hoarding training for 20 days. (+180 pA: injected 180 pA current.) **(C)** Summarized data showing firing rates of evoked action potentials in the groups as indicated in **(B)** (*n* = 8–10 neurons/group, control vs. hoarding: *F*_1,16_ = 4.66, *p* = 0.046; non-hoarding vs. hoarding: *F*_1,17_ = 4.89, *p* = 0.041; control vs. non-hoarding: *F*_1,15_ = 0.30, *p* = 0.592). **(D)** Schematic of viral injection and chemogenetic manipulation *in vivo*. **(E)** Behavioral effects of the chemogenetic activation of mPFC *GAD* neurons in non-hoarding and hoarding mice (*n* = 4–5 mice/group, control-mCherry: t_3_ = −0.22, *p* = 0.841; non-hoarding-hM3Dq: t_4_ = −0.10, *p* = 0.926; hoarding-hM3Dq: t_3_ = 5.12, *p* = 0.014). **(F)** Typical images of injection site and rAAV-*GAD67*-mCherry virus expression within the mPFC. [Scale bars: 500 μm (left), 10 μm (right).] **(G)** Schematic of viral injection and chemogenetic manipulation *in vivo*. **(H)** Behavioral effects of the chemogenetic inhibition of mPFC *GAD* neurons in non-hoarding and hoarding mice (*n* = 5–6 mice/group, control-mCherry: t_4_ = −0.39, *p* = 0.716; non-hoarding-hM4Di: t_4_ = −6.07, *p* = 0.004; hoarding-hM4Di: t_5_ = −0.22, *p* = 0.838). **(I)** Typical images of injection site and rAAV-*GAD67*-mCherry virus expression within the mPFC. [Scale bars: 500 μm (left), 10 μm (right).] Data are means ± SEM. ^*^p < 0.05, ^**^p < 0.01. ns, not significant. Two-way repeated-measures ANOVA with Bonferroni *post hoc* analysis for **(C)**; paired *t*-test for **(E,H)**.

Subsequently, we tracked the activity of mPFC GABAergic neurons by brain slice recording neurons infected with rAAV-*GAD67*-mCherry virus and verified the specificity of the virus, which was co-labeled with GABA antibody and not with glutamate antibody, indicating that the virus specifically identifies GABAergic neurons (Supplementary Figure 4). In response to step current injections, we found a decrease in the spike rate of mPFC mCherry+ neurons from hoarding mice compared with those of control or non-hoarding mice. These results suggest GABA neuronal activity is decreased in hoarding states (Figures 5A–C). Next, we evaluated whether activation of mPFC GABAergic neurons in hoarding mice could alleviate the hoarding-like behavior. The chemogenetic hM3Dq virus was injected in the mPFC, and on day 21 of hoarding training intraperitoneal injection of CNO to specifically activate GABAergic neurons in hoarding mice (Figure 5D), the excessive hoarding pattern was reversed (Figures 5E,F; Supplementary Figure 3C). Moreover, inhibition of GABAergic neurons significantly facilitated hoarding-like behavior in non-hoarding mice (Figures 5G–I; Supplementary Figure 3D). These results demonstrate that the mPFC GABAergic neurons are, at least in part, an inhibitory role that govern hoarding-like behavior.

**Figure 6:**
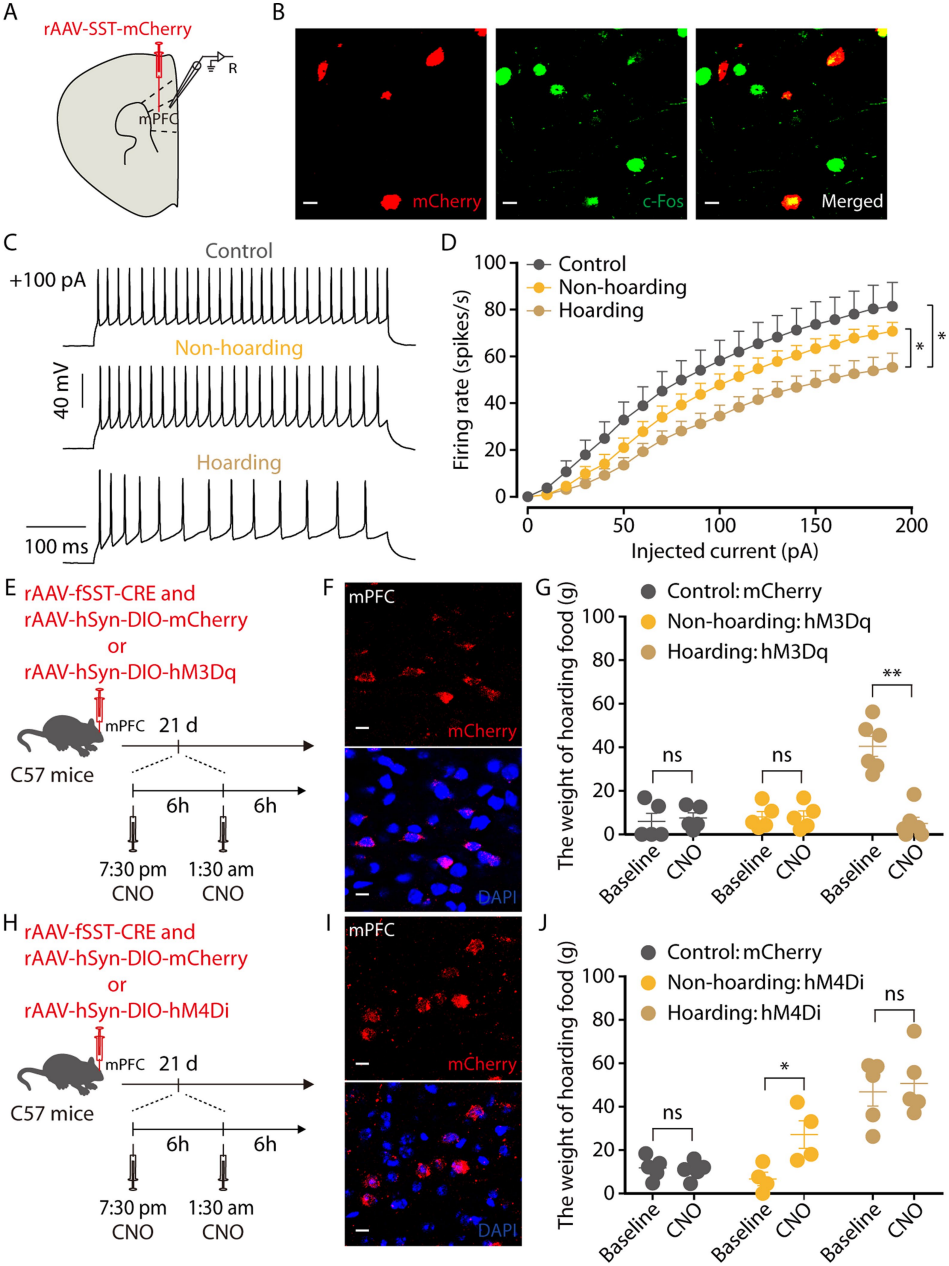
Somatostatin (SST)-expressing GABAergic neurons in the hoarding-like behavior mice. **(A)** Schematic diagram for rAAV-SST-mCherry virus injection and whole-cell recording in slices. **(B)** Example images of mCherry-positive neurons (red) merged with the c-Fos-positive neurons (green) in the mPFC. (Scale bars: 20 μm.) **(C)** Representative traces of voltage responses recorded from mCherry^+^ mPFC neurons in slices from mice treated with hoarding training for 20 days. (+100 pA: injected 100 pA current.) **(D)** Summarized data showing firing rates of evoked action potentials in the groups as indicated in **(C)** (*n* = 11–14 neurons/group, control vs. hoarding: *F*_1,23_ = 6.42, *p* = 0.019; non-hoarding vs. hoarding: F_1,25_ = 4.68, *p* = 0.040; control vs. non-hoarding: *F*_1,22_ = 1.51, *p* = 0.232). **(E)** Schematic of viral injection and chemogenetic manipulation *in vivo*. **(F)** Typical images of mCherry virus expression within the mPFC. (Scale bars: 10 μm.) **(G)** Behavioral effects of the chemogenetic activation of mPFC SST neurons in non-hoarding and hoarding mice (*n* = 5–6 mice/group, control-mCherry: t_4_ = −0.44, *p* = 0.681; non-hoarding-hM3Dq: t_4_ = −0.35, *p* = 0.743; hoarding-hM3Dq: t_5_ = 6.82, *p* = 0.001). **(H)** Schematic of viral injection and chemogenetic manipulation *in vivo*. **(I)** Typical images of mCherry virus expression within the mPFC. (Scale bars: 10 μm.) **(J)** Behavioral effects of the chemogenetic inhibition of mPFC SST neurons in non-hoarding and hoarding mice (*n* = 4–5 mice/group, control-mCherry: t_4_ = 0.45, *p* = 0.675; non-hoarding-hM4Di: t_3_ = −5.82, *p* = 0.010; hoarding-hM4Di: t_4_ = −0.79, *p* = 0.474). Data are means ± SEM. ^*^p < 0.05, ^**^p < 0.01. ns, not significant. Two-way repeated-measures ANOVA with Bonferroni *post hoc* analysis for **(D)**; paired *t*-test for **(G,J)**.

GABAergic neurons in the cerebral cortex have many different subtypes (Urban-Ciecko and Barth, 2016). By injection promoter-dependent viruses to co-label c-Fos expressing neurons in hoarding mice, we found that some of the mPFC c-Fos + neurons were somatostatin (SST)-expressing GABAergic neurons (Figures 6A,B), while a small percentage were parvalbumin (PV)-expressing GABAergic neurons (Supplementary Figure 5). We also verified the specificity of the virus, which was co-labeled with somatostatin antibody and not with glutamate antibody, indicating that the virus specifically identifies somatostatin neurons (Supplementary Figure 6). Using electrophysiology to record neuronal activity, we observed that the spike rate of mPFC SST neuronal was decreased in hoarding mice (Figures 6C,D), consistent with alterations in GABA neuronal activity. In addition, chemogenetic activation of mPFC SST neurons significantly reduced the weight of food hoarded by hoarding mice (Figures 6E–G), while chemogenetic inhibition of mPFC SST neurons significantly increased the weight of food hoarded by non-hoarding mice (Figures 6H–J). These results demonstrate that SST neurons, which account for approximately 30% of the total inhibitory neuron population (Lee et al., 2010), regulate hoarding-like behavior in mice. Notably, their synapses are nearly connected to glutamatergic neurons, providing inhibitory input to their activity (Fino and Yuste, 2011). This may explain why both mPFC’s glutamate and GABA neurons contribute to the exhibition of hoarding-like behavior.

## 4. Discussion

HD has three main features, clutter, difficulty to discard, and active and excessive acquisition (Mataix-Cols et al., 2010). People who hoard items often lack awareness of their serious behavior, causing great impairment and distress to themselves and their families, which is notoriously hard to treat (Steketee and Frost, 2003; Tolin et al., 2015). Reliable and efficient identification of relevant neural mechanisms of HD allow for a better understanding of their pathological behavior and appropriate therapeutic intervention. Most findings of animal hoarding emphasize that abnormal collection behavior is associated with dysfunction in the prefrontal areas (Kolb, 1974; Deacon et al., 2003; Deacon, 2006). Interestingly, this idea was validated in study of HD patients, who showed significantly increase prefrontal gray matter volume compared to OCD and healthy control groups (Yamada et al., 2018). In addition, our results also found that the medial prefrontal cortex neurons were hyperactivate in hoarding-like behavior mice. Hence, we propose that disfunction in the prefrontal regions responsible for decision making and emotion regulation could account for abnormal hoarding behavior.

As a new disease in DSM-5 (Regier et al., 2013), there is little research on neural mechanisms responsible for HD. Here, we established a mouse model of hoarding-like behavior by fasting, which is analogous to the excessive hoarding behavior of HD patients. Based on this, our findings in hoarding mice demonstrate that the mPFC region is involved in hoarding-like behavior, which is supported by a previous study that mPFC damage led to permanent deficit in hoarding food behavior (Kolb, 1974). In addition, we reveal that mPFC glutamatergic neurons activity is enhanced and GABAergic neurons activity is reduced in hoarding-like behavior (Supplementary Figure 7). And chemogenetic specifically regulated these neuronal activity which could modulate hoarding-like behavior accordingly. Furthermore, we found that GABAergic neurons, which are mainly SST neurons rather than PV neurons, are involved in hoarding-like behavior.

Maladaptation of the cortico-striato-thalamo-cortical (CSTC) circuits, including the dopamine and glutamate systems, is considered the basis for certain types of OCD symptoms (Ahmari et al., 2013; Sun et al., 2019). Although hoarding has been removed from the category of OCD, the results of our medial prefrontal cortex neuronal imbalance are consistent with this theory. Maybe, this is the reason why hoarding symptom was previously classified as a subtype of OCD symptoms. In summary, this study precisely dissects the functional relationship between mPFC neurons and hoarding-like behavior, providing new insights into the study of the treatment of human hoarding symptoms.

## Data availability statement

The original contributions presented in the study are included in the article/Supplementary material, further inquiries can be directed to the corresponding authors.

## Ethics statement

The animal study was reviewed and approved by Institutional Animal Care and Use Committee of Anhui Medical University.

## Author contributions

ZZhang and TS initiated and designed the research. YX, BW, YS, HL, and ZZhang performed the research and analyzed the data. ZZhang, QX, KW, and TS wrote the paper. All authors contributed to the article and approved the submitted version.

## Funding

This work was supported by National Natural Science Foundation of China (82001431) and Anhui Medical University (XJ201911 and 2019xkj007).

## Conflict of interest

The authors declare that the research was conducted in the absence of any commercial or financial relationships that could be construed as a potential conflict of interest.

## Publisher’s note

All claims expressed in this article are solely those of the authors and do not necessarily represent those of their affiliated organizations, or those of the publisher, the editors and the reviewers. Any product that may be evaluated in this article, or claim that may be made by its manufacturer, is not guaranteed or endorsed by the publisher.
