# Supplementary material for: Glutamatergic neurons and GABAergic neurons of medial prefrontal cortex control hoarding-like behavior
Source: Front Neurosci. 2023 May 11;17:1169927. doi: 10.3389/fnins.2023.1169927 (PMC10213654; doi:10.3389/fnins.2023.1169927)
Supplement: Supplementary file 1 [file Table_2.DOCX]

| Antibodies | | |
| --- | --- | --- |
| Rabbit Anti-c-Fos | Cell Signaling Technology | CAT # 2250 [1] |
| Rabbit Anti-GABA | Sigma Aldrich | CAT # A2052 [2, 3] |
| Rabbit Anti-glutamate | Sigma Aldrich | CAT # G6642 [2-4] |
| Rabbit Anti-somatostatin | Abcam | CAT # ab108456 [5] |
| Donkey anti-rabbit Alexa Fluor 488 | Abcam | CAT # ab150073 [6, 7] |
| Donkey anti-rabbit Alexa Fluor 647 | Abcam | CAT # ab150075 [8] |

1. Shin, S., et al., *Early adversity promotes binge-like eating habits by remodeling a leptin-responsive lateral hypothalamus-brainstem pathway.* Nat Neurosci, 2023. **26**(1): p. 79-91.

2. Yin, W., et al., *A Central Amygdala-Ventrolateral Periaqueductal Gray Matter Pathway for Pain in a Mouse Model of Depression-like Behavior.* Anesthesiology, 2020. **132**(5): p. 1175-1196.

3. Zhu, X., et al., *A Central Amygdala Input to the Parafascicular Nucleus Controls Comorbid Pain in Depression.* Cell Rep, 2019. **29**(12): p. 3847-3858 e5.

4. Sun, T., et al., *Basolateral amygdala input to the medial prefrontal cortex controls obsessive-compulsive disorder-like checking behavior.* Proc Natl Acad Sci U S A, 2019. **116**(9): p. 3799-3804.

5. Murayama, A.Y., et al., *The polymicrogyria-associated GPR56 promoter preferentially drives gene expression in developing GABAergic neurons in common marmosets.* Sci Rep, 2020. **10**(1): p. 21516.

6. Gunjigake, K., et al., *Interleukin-17A derived from mast cells contributes to fibrosis in gastric cancer with peritoneal dissemination.* Gastric Cancer, 2021. **24**(1): p. 31-44.

7. Kim, J. and J.A. Cooper, *Junctional Localization of Septin 2 Is Required for Organization of Junctional Proteins in Static Endothelial Monolayers.* Arterioscler Thromb Vasc Biol, 2021. **41**(1): p. 346-359.

8. Choi, H.J., et al., *Single-cell RNA sequencing of mitotic-arrested prospermatogonia with DAZL::GFP chickens and revealing unique epigenetic reprogramming of chickens.* J Anim Sci Biotechnol, 2022. **13**(1): p. 64.
