# Supplementary material for: Glutamatergic neurons and GABAergic neurons of medial prefrontal cortex control hoarding-like behavior
Source: Front Neurosci. 2023 May 11;17:1169927. doi: 10.3389/fnins.2023.1169927 (PMC10213654; doi:10.3389/fnins.2023.1169927)
Supplement: Supplementary file 2 [file Data_Sheet_1.docx]

Supplementary Material

Glutamatergic neurons and GABAergic neurons of medial prefrontal cortex control hoarding-like behavior

**Yujie Xiong^1, 4^, Beining Wang^1, 4^, Yunxia Shang^1^, Huan Liu^1^, Zihao Zhan^1^, Qi Xu^1^, Kai Wang^2^, Zhi Zhang^3^* and Tingting Sun^1^***

*** Correspondence:** Tingting Sun: sunting@mail.ustc.edu.cn

#
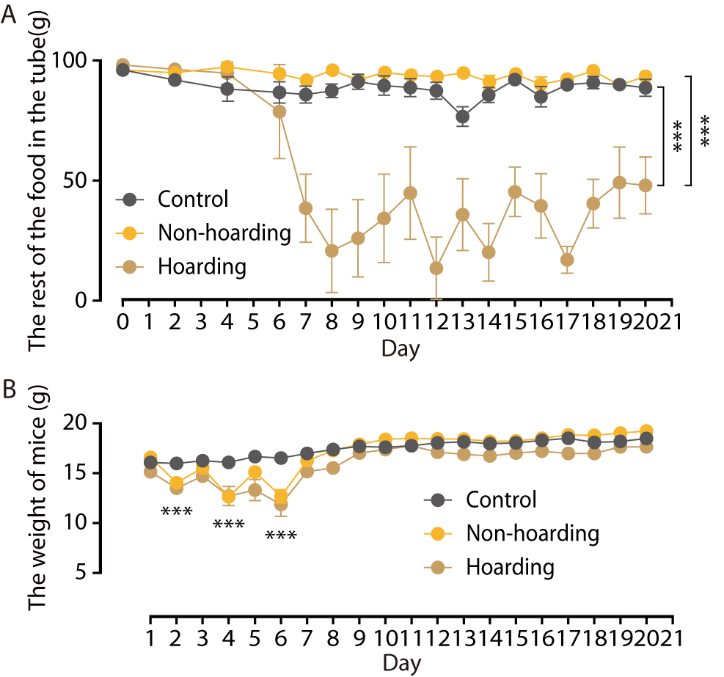
Supplementary Figures

**Supplementary Figure 1.** Additional supporting information related to Figure 1. (**A**) The rest of the food in the tube in mice treated with fasting 24 hours every other day three times (hoarding or non-hoarding) or without (control) (n = 5-12 mice/group, control vs. hoarding: F_1, 12_ = 71.58, P < 0.0001; non-hoarding vs. hoarding: F_1, 15_ = 132, P < 0.0001). (**B**) The weight of mice per day in the hoarding paradigm. [Day 2 (first 24h-fasting), control vs. hoarding: t_12_ = 4.83, P = 0.0004; day 4 (second 24h-fasting), control vs. hoarding: t_12_ = 4.97, P = 0.0003; day 6 (third 24h-fasting), control vs. hoarding: t_12_ = 5.13, P = 0.0002]. Data are means ± SEM. ***P < 0.001. Two-way repeated-measures ANOVA with Bonferroni *post hoc* analysis for (A); unpaired *t* test for (B).

**
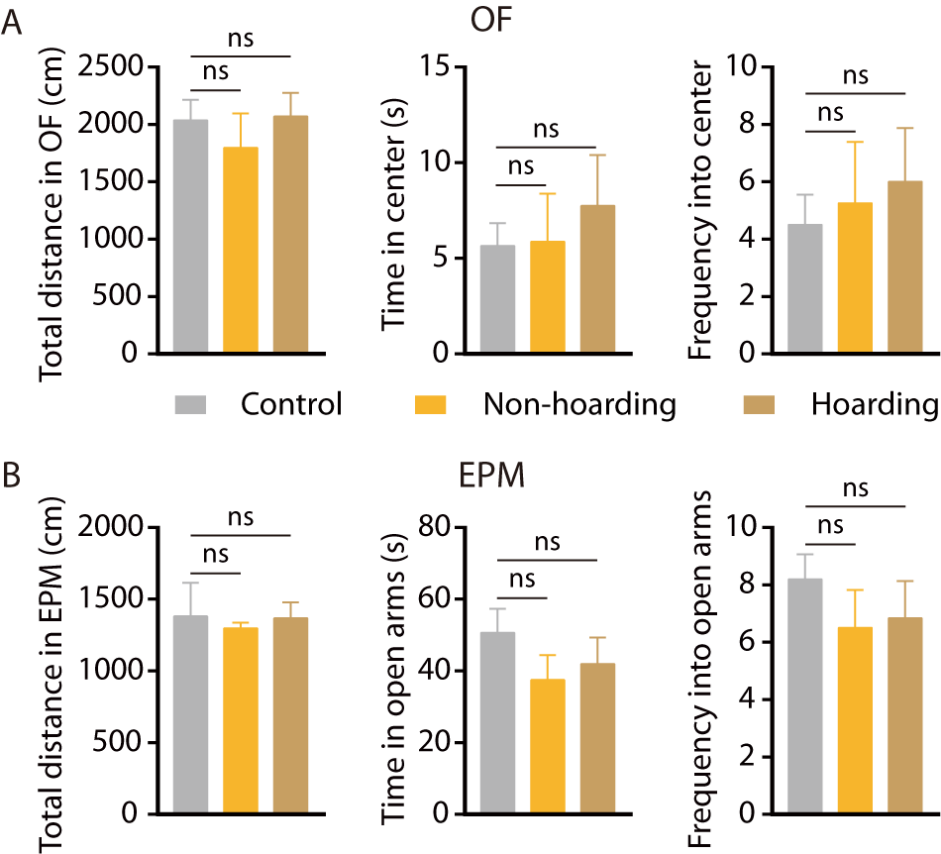
**

**Supplementary Figure 2.** No exhibit anxiety-like behavior in hoarding-like behavior mice. (**A**) Hoarding-like behavior mice showed no difference in the total distance traversed in the open field (OF), the time in center and the frequency into the center compared with control mice (n = 4-6 mice/group, total distance, control vs. non-hoarding: t_8_ = 0.73, P = 0.485; control vs. hoarding: t_10_ = -0.12, P = 0.903; time in center, control vs. non-hoarding: t_8_ = -0.09, P = 0.932; control vs. hoarding: t_10_ = -0.71, P = 0.492; frequency into center, control vs. non-hoarding: t_8_ = -0.35, P = 0.736; control vs. hoarding: t_10_ = -0.70, P = 0.503). (**B**) Hoarding-like behavior mice also showed no difference compared to the control group in the elevated plus maze (EPM) (n = 4-6 mice/group, total distance, control vs. non-hoarding: t_7_ = 0.31, P = 0.762; control vs. hoarding: t_9_ = 0.06, P = 0.957; time in open arms, control vs. non-hoarding: t_7_ = 1.35, P = 0.219; control vs. hoarding: t_9_ = 0.85, P = 0.415; frequency into open arms, control vs. non-hoarding: t_7_ = 1.12, P = 0.299; control vs. hoarding: t_9_ = 0.84, P = 0.425). Data are means ± SEM. ns, not significant. Unpaired *t* test for (A) and (B).

**
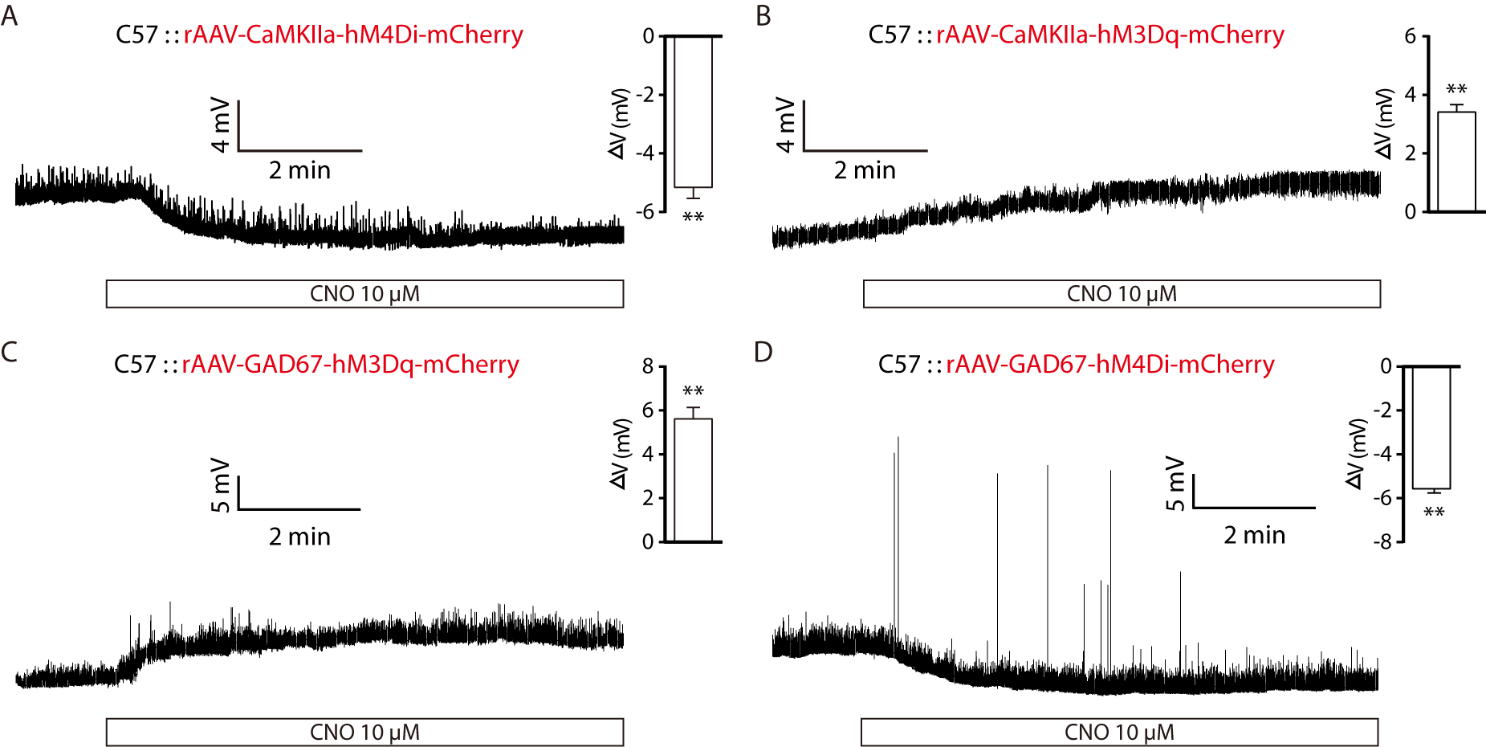
**

**Supplementary Figure 3.** The effects of CNO on synaptic transmission. (**A**) A representative trace (left) from a whole-cell current-clamp electrophysiological recording showing that bath application of CNO (10 μM) hyperpolarizes mPFC *CaMKII* neurons and statistics (right) showing the average magnitude of hyperpolarization (*n* = 3 neurons, *t*_2_ = 13.73, *P* = 0.005). (**B**) A representative trace (left) that bath application of CNO (10 μM) depolarizes mPFC *CaMKII* neurons and statistics (right) showing the average magnitude of depolarization (*n* = 3 neurons, *t*_2_ = -13.35, *P* = 0.006). (**C**) A representative trace (left) from a whole-cell current-clamp electrophysiological recording showing that bath application of CNO (10 μM) depolarizes mPFC *GAD* neurons and statistics (right) showing the average magnitude of depolarization (*n* = 3 neurons, *t*_2_ = -10.85, *P* = 0.008). (**D**) A representative trace (left) showing that bath application of CNO (10 μM) hyperpolarizes mPFC *GAD* neurons and statistics (right) showing the average magnitude of hyperpolarization (*n* = 3 neurons, *t*_2_ = 29.36, *P* = 0.001). Data are means ± SEM. ***P* < 0.01. Paired *t* test for (A), (B), (C), and (D).


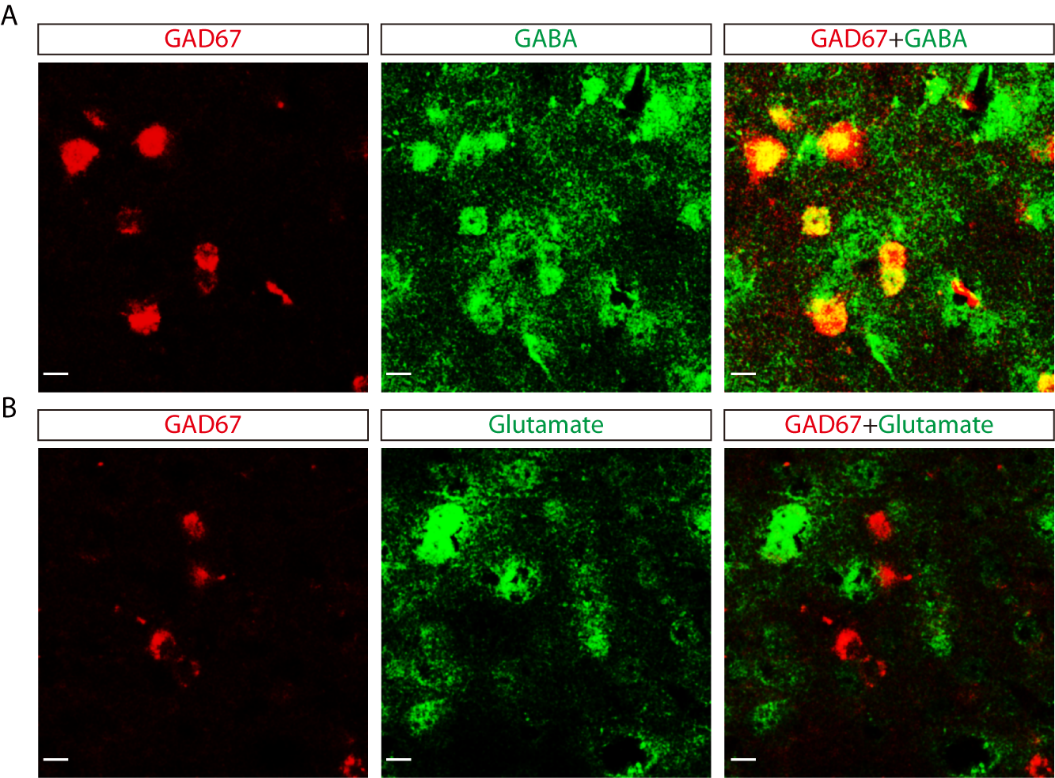


**Supplementary Figure 4.** Validation of the *GAD67* promoter virus specificity. (**A**) Example images of *GAD67* promoter virus-positive neurons (red) merged with the anti-GABA (green) in the mPFC. (Scale bars: 10 μm.) (**B**) Example images of *GAD67* promoter virus-positive neurons (red) did not merge with the anti-glutamate (green) in the mPFC. (Scale bars: 10 μm.)

**
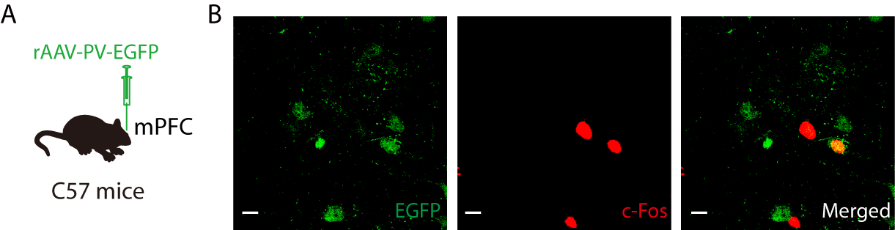
**

**Supplementary Figure 5.** Parvalbumin (PV)-expressing GABAergic neurons in the hoarding-like behavior mice. (**A**) Schematic diagram for rAAV-PV-EGFP virus injection. (**B**) Example images of EGFP-positive neurons (green) merged with the c-Fos-positive neurons (red) in the mPFC. (Scale bars: 20 μm.) Data are means ± SEM.


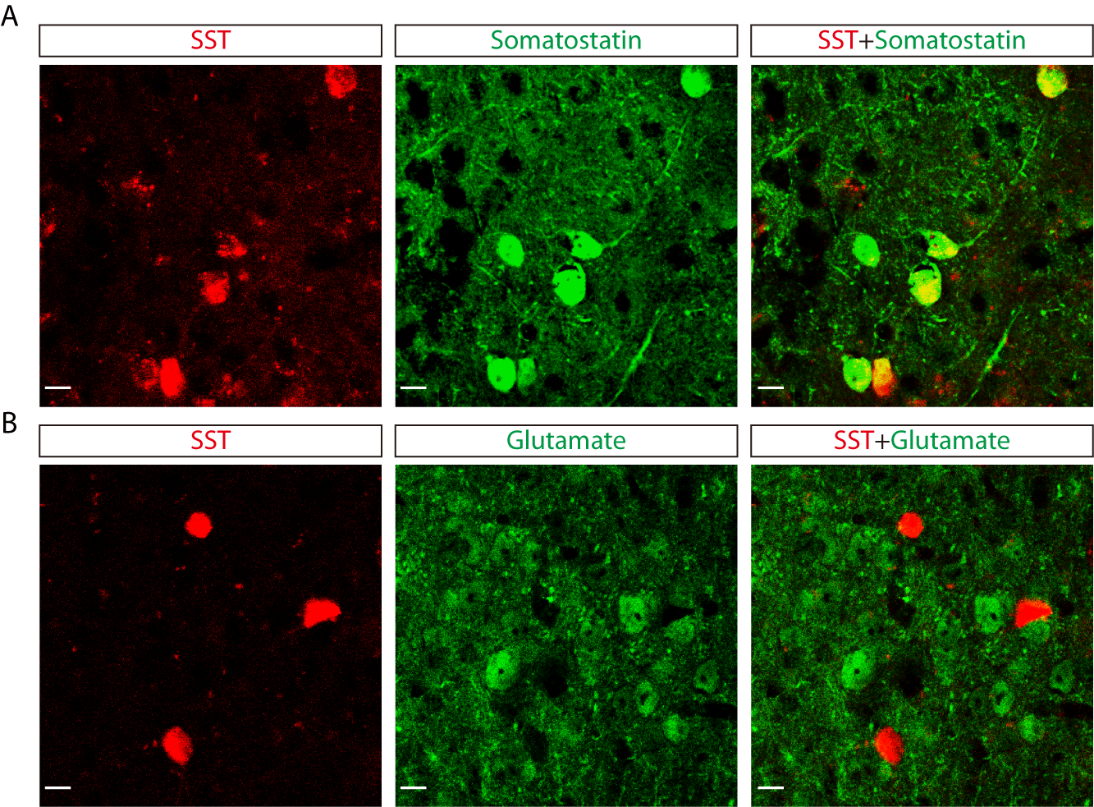


**Supplementary Figure 6.** Validation of the SST promoter virus specificity. (**A**) Example images of SST promoter virus-positive neurons (red) merged with the anti-somatostatin (green) in the mPFC. (Scale bars: 10 μm.) (**B**) Example images of SST promoter virus-positive neurons (red) did not merge with the anti-glutamate (green) in the mPFC. (Scale bars: 10 μm.)

**
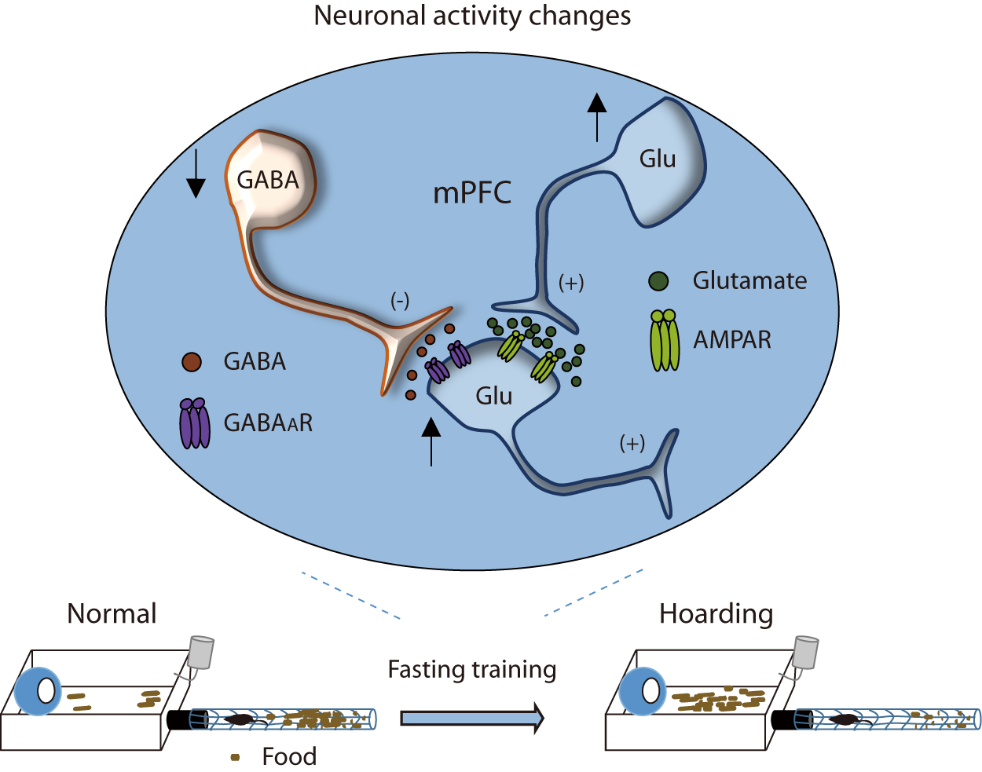
**

**Supplementary Figure 7.** The neuronal activity mechanisms underlying in hoarding-like behavior. In the hoarding-like behavior state, an increased excitatory process occurs within the decreased γ-aminobutyric acid–mediated (GABAergic) inputs (brown) that mediate disinhibition of the glutamatergic neurons (blue), and the increased excitatory transmitter input. The alterations in neuronal activity lead to the formation of hoarding-like behavior in mice. mPFC, medial prefrontal cortex; GABA, γ-aminobutyric acid; GABAAR, γ-aminobutyric acid type A receptor; Glu, glutamate; AMPAR, AMPA receptor.
